# Surgical repair and reconstruction of aortic arch in debakey type I aortic dissection: recent advances and single-center experience in the application of branched stent graft

**DOI:** 10.1186/s13019-017-0649-6

**Published:** 2017-10-03

**Authors:** Qian Zhang, Xiaochun Ma, Wenlong Zhang, Zhengjun Wang, Haizhou Zhang, Xiaofeng Zhang, Jian Song, Chengwei Zou

**Affiliations:** Department of Cardiovascular Surgery, Shandong Provincial Hospital affiliated to Shandong University, No.324 Jingwu Road, Shandong, 250021 People’s Republic of China

**Keywords:** Aortic dissection, Aortic arch, Branched stent graft

## Abstract

**Background:**

Aortic dissection (AD) represents a clinically uncommon aortic pathology which predicts a dismal prognosis if not promptly treated. In acute Debakey type I AD (ADIAD), aortic lesion extends from aortic root to even distal abdominal aorta among which aortic arch and its three main branches still remain a great surgical challenge for repair and reconstruction. Several decades have witnessed the painstaking efforts of cardiovascular surgeons across the globe for optimizing the surgical procedures, from total or hemi-arch replacement, “elephant trunk” technique to branched stent graft. However, operative mortality and morbidity still remain to be reduced and surgical strategy is to be advanced and simplified, particularly the repair and reconstruction of aortic arch and supra-aortic vessels.

**Methods:**

In this paper, we reviewed the relevant literature concerning recent advances in surgical intervention of aortic arch and summarized our opinions in the application of branched stent graft in ADIAD.

**Results:**

The operative strategy for acute Debakey type I aortic dissection still remain to be advanced and simplified, especially the repair and reconstruction of aortic arch and supra-aortic vessels. For selection of branched stent grafts, the anatomic features and pathological changes of diseased arch are the crucial factors for clinical decision making.

**Conclusions:**

Branched stent graft is potentially an effective alternative for the treatment of type I AD with diseased aortic arch and supra-aortic vessels. The selection of branched stent grafts still remains to be further discussed in large-scale studies in the future.

## Background

Aortic dissection (AD) is widely appreciated to be a catastrophic clinical event characteristic of intimal tear of aorta triggering influx of blood into the layers of aortic wall and denudation of the layers [1]. The disease has an incidence of approximately three per one hundred thousand every year in Europeans and it occurs at an annually increasing rate in Chinese population [2]. The disease is most frequently observed between the sixth and seventh decades of lifespan with an ascending proportion of middle-aged patients [3]. There exists a male predominance present in AD patients with a male to female ratio being approximately three to one [4]. The detailed mechanism of AD remains one of the major unresolved issues despite several well-documented risk factors that include hypertension, atherosclerosis, Marfan syndrome and deformity of bicuspid aortic valve [5]. AD predicts a dismal prognosis and half of the patients with Stanford type A AD fail to survive within the first twenty four hours after onset if not treated promptly, most of whom die of massive bleeding, cardiac tamponade, severe aortic regurgitation and acute heart failure [6]. In the year of 1965, Debakey and his colleagues initially proposed the classification of AD which has still been in clinical use to date [7]. Debakey type I AD is defined as original intimal flap situated in ascending aorta or aortic arch which extends distally to aortic arch, descending aorta and even distal end of abdominal aorta.

Urgent or elective surgical intervention is indicated by principle for acute Debakey type I AD (ADIAD), for the purpose of repair and reconstruction of the impaired aorta and aortic valve in time [8]. The very first successful attempt in the human history to treat AD by surgical means could be dated back to the year of 1965 by Debakey and his colleagues. More than fifty years from then has witnessed the remarkable achievements of surgical strategy for AD along with a great many advances in diagnosis, anesthesia, cardiopulmonary bypass (CPB), perioperative management and artificial implantation material [9]. For ADIAD, involvement of aortic arch and its three main branches complicates the disease and challenges the cardiovascular surgeons all over the world, to a large extent. The standard surgical procedures for ADIAD are contemporarily the ascending aortic arch replacement combined with hemi- or total- arch replacement and classical elephant trunk or stented elephant trunk technique [4]. Despite excellent curative effects, these procedures still confers relatively high surgical mortality and morbidity even after years of painstaking efforts endeavored by cardiovascular surgeons [10]. And these operations have been proven by decades of experience to be sophisticated, making the completion of surgery time-consuming and full of hardship [11]. In recent years, several groups across the globe, based on the classical procedures, have addressed this issue by proposing promising and novel surgical techniques—innovations which are of significant practical value [12–14]. Among these inventions, branched stent graft has gradually drawn attention for optimizing traditional surgery, though the criterion for selection of stent graft with different numbers of branches is still uncertain [10, 11, 13].

The primary purpose of this paper was to summarize recent advancements in surgical repair and reconstruction of aortic arch in ADIAD and in the meantime, share our experience of single-center concerning the application of branched stent graft in ADIAD.

## Recent surgical advances in acute Debakey type I aortic dissection

The operative principle for ADIAD lies in: 1) resection of the aortic wall where the intimal tear situates or occlusion of intimal tear; 2) implantation of befitting artificial blood vessels; 3) restoration of blood flow of true lumen; 4) repair or replacement of aortic valve and transplantation of coronary artery if necessary [9]. Specifically, how diseased aortic arch is surgically managed is varied in each patient with ADIAD. Thus surgical strategy tailored to individual patients, based on their own conditions, is recommended to obtain a favorable benefit to risk ratio. One of the key factors which should be given priority for consideration in personalized treatment is the anatomic details of damaged aortic arch and distal aorta. These anatomic details include the location and extent of intimal flap, the involvement of branched vessels of arch and distal aorta, and geometrical morphology of aorta and its branches [1].

### Hemi-arch replacement

Hemi-arch replacement is indicated for patients in which the intimal flap is restricted to the ascending aorta and the aortic arch has a diameter in the normal range without distal malperfusion. The procedure is literally described as the resection of ascending aorta and elimination of, in the greater curvature the aortic arch wall to the orifice of innominate artery, and in the lesser curvature the wall as much as possible [1]. Hemi-arch replacement has historically been the standard approach for ADIAD due to its noteworthy curative effects. Nevertheless, the long-term outcomes of patients undergoing hemi-arch replacement remains unsatisfactory because the limited graft replacement is incapable of eliminating residual false lumen or latent intimal tear present in aortic arch and distal aorta. Given the remnant aortic pathologies after hemi-arch replacement, this procedure may potentially places the patients who received the initial aortic surgery at excess risk of aortic dilation, dissection, rupture and thus reoperation. As a consequence, hemi-arch replacement requires long-term monitoring for patients to be prepared for recurrent dissection and secondary surgical management [15, 16]. A recent finding by Omura and his colleagues comparing the early and late outcomes of hemi-arch repair and total-arch repair in ADIAD demonstrated the similar five-year survivals between the two groups, but expectedly higher rates of distal aortic event of hemi-arch replacement group compared to total replacement group [17].

### Total-arch replacement

The indication of total-arch replacement for ADIAD includes: 1) extensive intimal tear in aortic arch, especially the involvement of supra-aortic orifices; and 2) aortic expansion. The Canadian Thoracic Aortic Collaborate (CTAC) recommends that an arch with a diameter of more than 45 mm should be given consideration for total-arch replacement. It is also recommended in ADIAD patients at younger age or with connective tissue disorders. Classical total-arch replacement comprises of two main types [17]. The first scenario is the connection of “island” of brachiocephalic vessels to a Dacron tube graft and the second one involves the anastomosis of three branch arteries and the four-branched prosthetic graft. A combination of total-arch replacement with elephant trunk technique once serves as the most advanced operation for ADIAD. However, total-arch replacement, to some extent, is still not the optimal operation for ADIAD. One reason lies in the complicated and sophisticated surgical skills challenging for cardiovascular surgeons to handle, such as anastomosis. For instance, the anastomosis of left subclavian artery and descending aorta are often remarkably difficult because of the deep surgical field. Second, duration of total-arch replacement remain lengthy, particularly the time course of circulatory arrest. The third rationale is the potential damage of recurrent laryngeal nerve that may occur in the operation. Another dilemma for surgeons is the difficulty of bleeding control during the operation and therefore the secondary postoperative massive bleeding [14, 18, 19].

### Traditional elephant trunk technique

As early as the year of 1983, Borst and his colleagues have first proposed to implant an artificial blood vessel into the aneurysm lumen in the proximal descending aorta through the incision of aortic arch-soft elephant trunk technique-in order to isolate the aneurysm lumen, reinforce the aortic wall and simplify the anastomosis of second-stage operation of distal descending aorta [20]. However, soft elephant trunk is not currently recommended for ADIAD, on account of the difficulty of insert of prostheses, limitation of support of aortic wall, disability of closure of intimal tear and restoration of true lumen, and possibility of deformation and twist of prostheses and secondary occlusion [21]. In the year of 1996, Keto and his team first reported the stented elephant trunk technique-also referred to as frozen elephant trunk technique-in which a self-expanding circular stent replaced the soft prostheses to overcome the disadvantages of soft elephant trunk technique [22]. Subsequently, more aggressive operative repair and reconstruction of arch was proposed by Sun and his colleagues. Sun’s procedure, namely the stented elephant trunk combined with four-branched graft replacement, is contemporarily the standard procedure which has won widespread recognition in the treatment of ADIAD [14, 23]. And Sun’s procedure is indicated in the patients with ADIAD: 1) dissection involving the supra-aortic vessels; 2) primary intimal tear located in the aortic arch and proximal descending aorta; 3) dilation aortic arch, supra-arch vessels or proximal descending aorta; 4) Marfan syndrome and other connective tissue disorders [24, 25].

### Hybrid four-branched frozen elephant trunk

In 2006, Herold et al. described an E-vita open hybrid prosthesis made out of a proximal polyester tube graft and a distal self-expandable nitinol stent graft [26–28]. Recently Shrestha and his group provided the description of a novel four-branched frozen elephant trunk prosthesis as well as a single-center experiences of first group of patients undergoing the implantation with ADIAD. This new technique is in essence the fusion of canonical elephant trunk and conventional four-branched prosthesis graft. In particular, the proximal unstented and distal stented parts are available with various size and there exists a sewing collar between the two parts which simplified the anastomosis. The graft comprises of a self-expandable stent coated with polyester vascular graft fabric and the four-branched graft consists of three sidearms of supra-aortic vessels and one sidearm for CPB. The implantation of this prosthesis yielded excellent outcomes and aortic remodeling in ADIAD [29].

### Branched stent graft

Nearly from the year of 2010 to date, Chen and his colleagues have consecutively reported the application of the triple-branched stent graft in the treatment of ADIAD and also the improved version of the stented grafts. The triple-branched stent graft includes a main graft and three sidearms with different diameters which consist of the self-expanding nitinol stent covered with polyester fabric. The proximal part of the main graft is a stent-free Dacron tube previously designed for anastomosis. The triple-branched stent graft can be inserted into the true lumen of aortic arch and descending aorta under direct vision with the aid of a transverse incision of distal ascending aorta [11, 13, 19, 30, 31]. The authors have demonstrated this stented graft to be an effective and simplified alternative for hemi- or total- arch replacement in ADIAD [11, 30]. However, the anatomic features of diseased aortic arch are the determinant for implantation of the stented graft which include the diameters of the arch and its branched vessels, the distance between the supra-aortic vessels, the curvature of arch and the direction of arch vessels [13, 32]. Thus the first generation of triple-branched stent graft is not generalized to each patient in the clinical setting.

To resolve this issue, Chen and his team reported the updated version of the stented graft-the self-adaptive triple-branched stent graft. Its main differences with the first generation lies in that the proximal portion of the main graft is stent-free, the distance between two neighboring sidearms was larger than that between two neighboring supra-aortic vessels of Chinese patients, and the diameter of main tube is bigger than normal ranges of Chinese ethnicity. The second generation of stented graft was positioned with the help of an arch longitudinal incision, after which an independent arch stent was placed in the proximal portion of the main graft. Due to the soft fabric of the proximal main tube, the prosthesis can easily fit the anatomic features of different patients [13, 32].

Not confined to the triple-branched stent graft, the stented grafts with one or two branches for supra-aortic vessels are also available for clinical application in ADIAD [10, 26]. However, there is no consensus that has gained widespread recognition in the selection of stented prosthesis with different branches in ADIAD. And we will further discuss this issue later in this paper.

Compared to “the frozen elephant trunk” technique, implantation of branched stent comprises the advantages that include: 1) greatly simplifying the surgery in comparison with the complicated and sophisticated surgical skills challenging for cardiovascular surgeons to handle in the “the frozen elephant trunk” (such as bleeding control and anastomosis); 2) reducing the operation time, particularly the time course of circulatory arrest; 3) avoiding the potential damage of recurrent laryngeal nerve that may occur in the operation.

### Branch-first arch replacement

Despite the refinements in surgical and perfusion techniques over several decades, the incidence of cerebral injury remain high in the surgery of the aortic arch contemporarily with the approaches that include hypothermia and antegrade and/or retrograde cerebral perfusion. Galvin and his team have recently highlighted the application of“branch-first” continuous perfusion aortic arch replacement in ADIAD for the purpose of improved cerebral outcomes in particular. The graft is essentially a modified triple-branched Dacron graft that harbors an additional limb for antegrade cerebral perfusion after each branch vessel anastomosis. Thus this feature saves the need for right axillary cannulation to sustain the blood supply for brain. In the process of“branch-first” aortic reconstruction, the innominate artery, left common carotid artery and left subclavian artery were connected to the branches of trifurcated graft in sequence and the blood perfusion is resumed after the completion of anastomosis of each vessels. Then the anastomosis of arch graft to the distal aorta finishes and connection of the trifurcated graft to the ascending graft continues without the interruption of perfusion [33–36].

### Selection of branched stent graft in ADIAD

#### Patient characteristics and perioperative data

From 2011 to 2015, 46 consecutive patients with ADIAD were recruited from the Department of Cardiovascular Surgery of the Provincial Hospital Affiliated Shandong University, and were hospitalized for implantation of branched stent graft. Amongst these patients, 32 were male and 14 were female, with an average age of 48 (range, 28–63 years). Among these patients, 40, 5 and 1 patients received the implantation of one-, triple- and two-branched stent graft, respectively. The patient characteristics and perioperative data were summarized in the Table 1. This study was approved by the ethics committee of the Provincial Hospital Affiliated Shandong University. Formal written consent was obtained from each patient or legal representative. The included patients were selected to undergo the implantation of stented graft with different numbers of branches.

**Table 1:** Patient characteristics and perioperative data

Variables	Included Subjects
Preoperative data	
Patients, no. (%)	46
Males, no. (%)	32 (69.6%)
Age, year	48 (28–63)
Marfan syndrome, no. (%)	2 (4.3%)
Renal insufficiency, no. (%)	2 (4.3%)
Aortic regurgitation, no. (%)	5 (10.9%)
Cerebral ischemia, no. (%)	3 (6.5%)
Supra-aortic vessels involved, no. (%)	7 (15.2%)
Operative data	
Operation time, min	355.3 ± 38.6
Cardiopulmonary bypass time, min	168.7 ± 23.1
Cardiac ischemia time, min	78.1 ± 18.9
Circulatory arrest or selective cerebral perfusion time, min	36.8 ± 8.3
One-branched stent graft, no. (%)	40 (87.0%)
Two-branched stent graft, no. (%)	1 (2.2%)
Triple-branched stent graft, no. (%)	5 (10.9%)
Postoperative data	
Postoperative drainage fluid, mL	783.6 ± 68.7
Hospitalization time, day	19.5 ± 6.1
ICU time, day	3.9 ± 1.2

#### Operative procedures

A median sternotomy was performed under general anesthesia and total cardiopulmonary bypass (CPB) was established by positioning an arterial cannula in the right axillary artery and two venous cannulas through right atrium. After carefully dissecting the ascending aorta, aortic arch and supra-arch vessels, a longitudinal incision was performed subsequent to the clamping of the ascending aorta. The cold blood cardioplegia was used by perfusion through the left and right coronary arteries for myocardial protection. Repair and reconstruction of the aortic arch was performed under profound hypothermic, circulatory arrest and selective antegrade cerebral perfusion. During the core cooling, concomitant cardiac procedures were performed if necessary, such as aortic valve repair or replacement, sinus reconstruction, and root replacement.

**Fig. 1:**
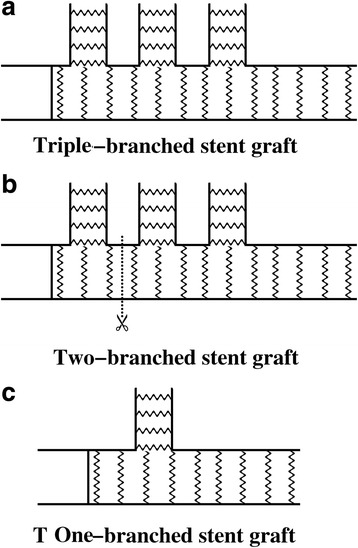
The triple-, two- and one-branched stent graft. **a** The triple-branched stent graft includes a main graft and three sidearms with different diameters which consist of the self-expanding nitinol stent covered with polyester fabric. The proximal part of the main graft is a stent-free Dacron tube previously designed for anastomosis. **b** The two-branched stent graft acts as a trimmed version of triple-branched stent graft, with the branch for innominate artery cut off. **c** The one-branched stent graft comprises of a main graft and only one sidearm for left subclavian artery

The patients were cooled till the nasopharyngeal temperature dropped to 15 °C. Then the systemic circulation was arrested and selective anterograde cerebral perfusion via the right axillary artery was established by clamping the innominate artery. The clamping of the ascending aorta was subsequently removed and the aortic arch was opened with the incision from the ascending aorta to the base of innominate artery. The location and extent of intimal tear and dissection in aortic arch, supra-aortic vessels and proximal descending aorta were explored in order to determine the choice of branched stent graft (Fig. 1). Another crucial factor was the anatomic features of the diseased arch as above-mentioned. The main graft of the branched stent graft was implanted into the true lumen of aortic arch and proximal descending aorta, after which the sidearm tube grafts were inserted in sequence and correctly deployed. More specifically, for triple-branched stent graft, the ascending aorta was reconstructed using a straight or single-branched Dacron tube graft which was connected to the aortic root and stent graft respectively by continuous anastomosis. For single-branched stent graft implantation in which the stented graft was inserted into proximal descending aorta and only left subclavian artery, the combined total-arch replacement was performed in the meantime for ascending aorta and arch replacement as described above. In the case of two-branched stent graft, a trimmed triple-branched stent graft was in fact applied in which the stented prosthesis was deployed into the proximal descending aorta, left subclavian artery and left common carotid artery with the branch for innominate artery cut off. And the patients underwent the simultaneous hemi-arch and ascending aorta replacement with the use of a single-branched Dacron tube graft.

#### Selection of branched stent graft-our opinions

**Fig. 2:**
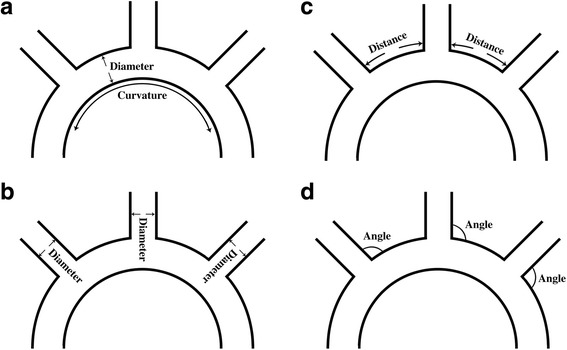
The anatomic features of diseased arch worth consideration for selection of branched stent grafts. **a** The diameter and curvature of the arch. **b** The diameters of arch vessels. **c** The distance between arch vessels. **d** The angles between arch and arch vessels

**Fig. 3:**
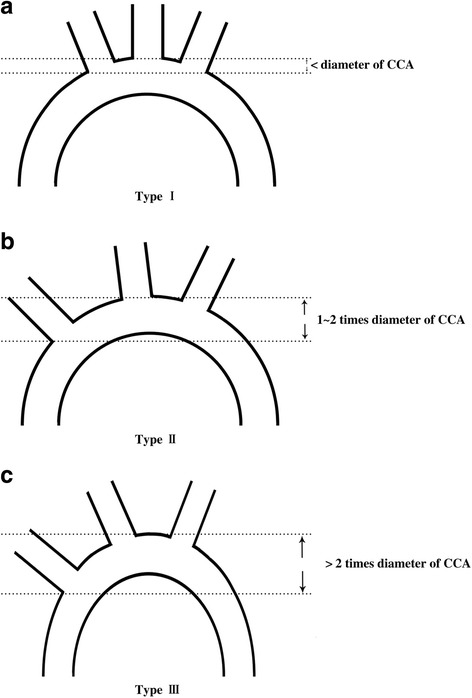
Three types of aortic arch. Aortic arch can be divided into three types according to the ratio between diameter of common carotid artery (CCA) to the distance between the horizontal line through the top of arch and the horizontal line through the orifice of innominate artery: **a** type I, less than 1; **b** type II, between 1 to 2, and **c** type III, more than 2

1. Among all the factors worth consideration for selection of branched stent grafts, the anatomic features of diseased arch should be in the very first place. These anatomic characteristics include: A) the diameter and curvature of the arch; B) the diameters of arch vessels; C) the distance between arch vessels; and D) the angles between arch and arch vessels (Fig. 2). For instance, it is inappropriate to implant the triple-branched stent graft in patients with innominate artery and left common carotid artery which originate from one orifice or have no interval between each other. Under such circumstances, we applied Sun’s procedure or transplantation of one-branched stent graft instead of triple-branched stent graft. And if the angle between left subclavian artery and arch is relatively limited, the patients who received the implantation of one-branched stent graft will take a higher risk of graft buckling, and the effective cross-sectional area for blood flow of left subclavian artery is also compromised. Additionally, the most common complications of branched stent graft implantation are migration and endoleak due to the selection of stented grafts with too small sizes. To avoid this, Chen and his colleagues recommended that the diameters of the aortic arch and arch branches should be 10% to 20% smaller than those of the corresponding stent grafts [13, 19]. Conversely, the stented grafts with oversize diameters will stress the aortic wall and even lead to a catastrophic rupture of aorta. Besides, aortic arch can be divided into three types according to the ratio between diameter of common carotid artery (CCA) to the distance between the horizontal line through the top of arch and the horizontal line through the orifice of innominate artery: type I, less than 1; type II, between 1 to 2, and type III, more than 2 (Fig. 3). We proposed that type I aortic arch is relatively more suitable for implantation of triple-branched stent graft while for the patients with type II and III arch, Sun’s procedure or transplantation of one-branched stent graft might be more befitting choice in ADIAD.
2. Another key factor for branched stent graft implantation relies on the situations of pathological changes of diseased aortic arch. We recommend not to implant the branched stent graft under the circumstances of the aortic lesions below: A) aortic aneurysm in aortic arch, supra-arch vessels or proximal descending aorta; B) evident dilation or widening of aortic arch, supra-arch vessels or proximal descending aorta; C) obvious edema, ischemia and degeneration of aortic arch, supra-arch vessels or proximal descending aorta; D) concomitant carotid artery or subclavian artery disease.
  On the premise of (1) and (2):
3. Compared to triple- and one-branched stented graft, two-branched stented graft has seldomly been reported in retrospective observational studies and we once used a trimmed triple-branched stent graft (with the branch for innominate artery cut off) as an alternative. We proposed that two-branched stented graft could be applied in the cases in which the intimal tear is situated in the opening of innominate artery whereas no intimal tear is found in the other part of arch, supra-arch vessels and proximal descending aorta.
4. Placement of a triple-branched stent graft has been proved to be more challenging than that of a one-branched stent graft because what we have discussed in [1]. Although the first generation of triple-branched stent has various types with different parameters, they still fail to satisfy all the individuals. We recommend the triple-branched stent graft in the cases in which no intimal tear is located in the arch, arch branch vessels and proximal descending aorta (or intimal tear is restricted to the lesser curvature with the other parts of arch and supra-arch vessels not involved).
5. One-branched stent graft has recently been recognized in ADIAD and it has wider indication than triple-branched stent. In our pool of cases, we also applied one-branched stent in the patients in whom the left subclavian artery was retained while the other parts of arch, innominate artery and left common carotid artery were removed because of aneurysm, edema and dilation.

## Conclusions

The operative strategy for acute Debakey type I aortic dissection still remain to be advanced and simplified, especially the repair and reconstruction of aortic arch and supra-aortic vessels. For selection of branched stent grafts, the anatomic features and pathological changes of diseased arch are the crucial factors for clinical decision making.

## Abbreviations

AD: Aortic dissection
ADIAD: Acute Debakey type I AD
CCA: Common carotid artery
CPB: Cardiopulmonary bypass
CTAC: The Canadian Thoracic Aortic Collaborate

## References

[1] El-Hamamsy I, Ouzounian M, Demers P, McClure S, Hassan A, Dagenais F (2016). State-of-the-Art Surgical Management of Acute Type A Aortic Dissection. Can J Cardiol.

[2] Wang W, Duan W, Xue Y, Wang L, Liu J, Yu S (2014). Clinical features of acute aortic dissection from the Registry of Aortic Dissection in China. J Thorac Cardiovasc Surg.

[3] Criado FJ (2011). Aortic dissection: a 250-year perspective. Tex Heart Inst J.

[4] Nienaber CA, Clough RE (2015). Management of acute aortic dissection. Lancet.

[5] Xiao ZY, Yao CL, Gu GR (2016). Research update on the pathogenesis of aortic dissection. Zhonghua Xin Xue Guan Bing Za Zhi.

[6] Tolis G, Sundt Iii TM (2016). Contemporary insights into the management of type A aortic dissection. Expert Rev Cardiovasc Ther.

[7] Debakey ME, Henly WS, Cooley DA, Morris GC, Crawford ES, Beall AC (1965). Surgical Management of Dissecting Aneurysms of the Aorta. J Thorac Cardiovasc Surg.

[8] Kamalakannan D, Rosman HS, Eagle KA. Acute aortic dissection. Crit Care Clin. 2007;23(4):779–800, vi. Epub 2007/10/30 10.1016/j.ccc.2007.07.002 17964363

[9] Hussain ST, Svensson LG (2016). Surgical techniques in type A dissection. Ann Cardiothor Surg.

[10] Chen LW, Dai XF, Yang GF, Zhang GC, Cao H, Wang QM (2010). Open-branched stent graft placement makes total arch replacement easier for acute type a aortic dissection. Ann Thorac Surg.

[11] Dai XF, Chen LW, Wu XJ, Dong Y, Wang QM (2015). Total Aortic Arch Reconstruction With Triple-Branched Stent Graft or Hemiarch Replacement for Acute Debakey Type I Aortic Dissection: Five-Years Experience With 93 Patients. J Card Surg.

[12] Shrestha M, Kaufeld T, Beckmann E, Fleissner F, Umminger J, Abd Alhadi F, et al. Total aortic arch replacement with a novel 4-branched frozen elephant trunk prosthesis: Single-center results of the first 100 patients. J Thorac Cardiovasc Surg. 2016;152(1):148–159 e1. Epub 2016/05/12 10.1016/j.jtcvs.2016.02.077 27167026

[13] Chen LW, Wu XJ, Dai XF, Liao DS, Li C, Wang QM, et al. A self-adaptive triple-branched stent graft for arch repair during open type A dissection surgery. J Thorac Cardiovasc Surg. 2015;149(5):1278–1283 e1. Epub 2015/01/20 10.1016/j.jtcvs.2014.11.079 25598526

[14] Sun L, Qi R, Chang Q, Zhu J, Liu Y, Yu C (2009). Surgery for acute type A dissection with the tear in the descending aorta using a stented elephant trunk procedure. Ann Thorac Surg.

[15] Fattouch K, Sampognaro R, Navarra E, Caruso M, Pisano C, Coppola G (2009). Long-term results after repair of type a acute aortic dissection according to false lumen patency. Ann Thorac Surg.

[16] Kimura N, Tanaka M, Kawahito K, Yamaguchi A, Ino T, Adachi H (2008). Influence of patent false lumen on long-term outcome after surgery for acute type A aortic dissection. J Thorac Cardiovasc Surg.

[17] Omura A, Miyahara S, Yamanaka K, Sakamoto T, Matsumori M, Okada K (2016). Early and late outcomes of repaired acute DeBakey type I aortic dissection after graft replacement. J Thorac Cardiovasc Surg.

[18] Park KH, Lim C, Choi JH, Chung E, Choi SI, Chun EJ (2009). Midterm change of descending aortic false lumen after repair of acute type I dissection. Ann Thorac Surg.

[19] Chen LW, Dai XF, Zhang GC, Lu L. Total aortic arch reconstruction with open placement of triple-branched stent graft for acute type A dissection. J Thorac Cardiovasc Surg. 2010;139(6):1654–1655 e1. Epub 2010/01/16 10.1016/j.jtcvs.2009.10.022 20074749

[20] Borst HG, Walterbusch G, Schaps D (1983). Extensive aortic replacement using “elephant trunk” prosthesis. Thorac Cardiovasc Surg.

[21] Crawford ES, Coselli JS, Svensson LG, Safi HJ, Hess KR (1990). Diffuse aneurysmal disease (chronic aortic dissection, Marfan, and mega aorta syndromes) and multiple aneurysm. Treatment by subtotal and total aortic replacement emphasizing the elephant trunk operation. Ann Surg.

[22] Kato M, Ohnishi K, Kaneko M, Ueda T, Kishi D, Mizushima T (1996). New graft-implanting method for thoracic aortic aneurysm or dissection with a stented graft. Circulation.

[23] Sun L, Qi R, Chang Q, Zhu J, Liu Y, Yu C (2008). Surgery for marfan patients with acute type a dissection using a stented elephant trunk procedure. Ann Thorac Surg.

[24] Sun LZ, Ma WG, Zhu JM, Zheng J, Liu YM, Ziganshin BA (2013). Sun's procedure for chronic type A aortic dissection: total arch replacement using a tetrafurcate graft with stented elephant trunk implantation. Ann Cardiothorac Surg.

[25] Sun L, Qi R, Zhu J, Liu Y, Zheng J (2011). Total arch replacement combined with stented elephant trunk implantation: a new "standard" therapy for type a dissection involving repair of the aortic arch?. Circulation.

[26] Shimamura K, Kuratani T, Matsumiya G, Shirakawa Y, Takeuchi M, Takano H (2009). Hybrid endovascular aortic arch repair using branched endoprosthesis: the second-generation "branched" open stent-grafting technique. J Thorac Cardiovasc Surg.

[27] Jakob H, Tsagakis K (2013). International E-vita open registry. Ann Cardiothorac Surg.

[28] Di Bartolomeo R, Cefarelli M, Folesani G, Di Eusanio M (2013). Frozen elephant trunk surgery using the Vascutek Thora-flex hybrid prosthesis. Ann Cardiothorac Surg.

[29] Shrestha M, Pichlmaier M, Martens A, Hagl C, Khaladj N, Haverich A (2013). Total aortic arch replacement with a novel four-branched frozen elephant trunk graft: first-in-man results. Ann Cardiothorac Surg.

[30] Lu S, Yang S, Lai H, Zheng J, Hong T, Sun X (2016). Open aortic arch reconstruction for acute type a aortic dissection: a single-center experience with 267 consecutive patients. J Cardiothorac Surg.

[31] Chen LW, Lu L, Dai XF, Wu XJ, Zhang GC, Yang GF (2014). Total arch repair with open triple-branched stent graft placement for acute type A aortic dissection: experience with 122 patients. J Thorac Cardiovasc Surg.

[32] Chen LW, Wu XJ, Dai XF, Lu L, Liao DS, Li C (2014). Total arch repair for acute type A aortic dissection with open placement of a modified triple-branched stent graft and the arch open technique. J Cardiothorac Surg.

[33] Galvin SD, Perera NK, Matalanis G (2016). Surgical management of acute type A aortic dissection: branch-first arch replacement with total aortic repair. Ann Cardiothorac Surg.

[34] Matalanis G, Perera NK, Galvin SD (2015). Aortic arch replacement without circulatory arrest or deep hypothermia: the "branch-first" technique. J Thorac Cardiovasc Surg.

[35] Galvin SD, Matalanis G (2013). Continuous perfusion "Branch-first" aortic arch replacement: a technical perspective. Ann Cardiothorac Surg.

[36] Matalanis G, Galvin SD (2013). "Branch-first" continuous perfusion aortic arch replacement and its role in intra-operative cerebral protection. Ann Cardiothorac Surg.

